# Whole-exome sequencing and bioinformatic analyses revealed differences in gene mutation profiles in papillary thyroid cancer patients with and without benign thyroid goitre background

**DOI:** 10.3389/fendo.2022.1039494

**Published:** 2023-01-04

**Authors:** Zing Hong Eng, Mardiaty Iryani Abdullah, Khoon Leong Ng, Azlina Abdul Aziz, Nurul Hannis Arba’ie, Nurullainy Mat Rashid, Sarni Mat Junit

**Affiliations:** ^1^ Department of Molecular Medicine, Faculty of Medicine, Universiti Malaya, Kuala Lumpur, Malaysia; ^2^ Department of Biomedical Science, Kulliyyah of Allied Health Sciences, International Islamic University Malaysia, Kuantan, Pahang, Malaysia; ^3^ Department of Surgery, Faculty of Medicine, Universiti Malaya, Kuala Lumpur, Malaysia

**Keywords:** benign thyroid goitre, whole-exome sequencing, thyroid tumourigenesis, single nucleotide variants, papillary thyroid cancer

## Abstract

**Background:**

Papillary thyroid cancer (PTC) is the most common thyroid malignancy. Concurrent presence of cytomorphological benign thyroid goitre (BTG) and PTC lesion is often detected. Aberrant protein profiles were previously reported in patients with and without BTG cytomorphological background. This study aimed to evaluate gene mutation profiles to further understand the molecular mechanism underlying BTG, PTC without BTG background and PTC with BTG background.

**Methods:**

Patients were grouped according to the histopathological examination results: (i) BTG patients (n = 9), (ii) PTC patients without BTG background (PTCa, n = 8), and (iii) PTC patients with BTG background (PTCb, n = 5). Whole-exome sequencing (WES) was performed on genomic DNA extracted from thyroid tissue specimens. Nonsynonymous and splice-site variants with MAF of ≤ 1% in the 1000 Genomes Project were subjected to principal component analysis (PCA). PTC-specific SNVs were filtered against OncoKB and COSMIC while novel SNVs were screened through dbSNP and COSMIC databases. Functional impacts of the SNVs were predicted using PolyPhen-2 and SIFT. Protein-protein interaction (PPI) enrichment of the tumour-related genes was analysed using Metascape and MCODE algorithm.

**Results:**

PCA plots showed distinctive SNV profiles among the three groups. OncoKB and COSMIC database screening identified 36 tumour-related genes including *BRCA2* and *FANCD2* in all groups. *BRAF* and 19 additional genes were found only in PTCa and PTCb. “Pathways in cancer”, “DNA repair” and “Fanconi anaemia pathway” were among the top networks shared by all groups. However, signalling pathways related to tyrosine kinases were the most significantly enriched in PTCa while “Jak-STAT signalling pathway” and “Notch signalling pathway” were the only significantly enriched in PTCb. Ten SNVs were PTC-specific of which two were novel; *DCTN1* c.2786C>G (p.Ala929Gly) and *TRRAP* c.8735G>C (p.Ser2912Thr). Four out of the ten SNVs were unique to PTCa.

**Conclusion:**

Distinctive gene mutation patterns detected in this study corroborated the previous protein profile findings. We hypothesised that the PTCa and PTCb subtypes differed in the underlying molecular mechanisms involving tyrosine kinase, Jak-STAT and Notch signalling pathways. The potential applications of the SNVs in differentiating the benign from the PTC subtypes requires further validation in a larger sample size.

## 1 Introduction

Abnormal focal growth of thyroid cells resulting in thyroid nodules are very common in the general population. Although the majority of them are diagnosed as benign thyroid goitre (BTG), malignancies occur in 5% to 10% of nodules (1). Thyroid cancer, the most prevalent endocrine malignancy (2), is in the top ten of the most common cancer types in the world’s female population, including Malaysia (3). Papillary thyroid cancer (PTC) is thyroid follicular cell-derived, constituting approximately 80% of all thyroid cancer cases (4). Mutations that trigger oncogenic activation of mitogen-activated protein kinase (MAPK) signalling pathway such as the *BRAF* ^V600E^ mutation are frequently linked to many malignancies including melanoma, colorectal cancer, and PTC (5).

Fine-needle aspiration cytology (FNAC) is a standard pre-operative, minimally invasive procedure to determine thyroid nodule malignancy status. However, up to one-fourth of the cases usually falls into the indeterminate Bethesda categories III and IV (6). Malignancy status is usually confirmed by histopathological examination (HPE) of thyroid tissue samples following partial or total thyroidectomy. HPE is the gold standard for thyroid nodule diagnosis, and yet only 40% of the indeterminate nodules were confirmed to be malignant after HPE (6, 7). Particularly, some of the confirmed PTC through HPE biopsies were found to have a BTG cytomorphological structure. Whether these PTCs are unique PTC subtypes, or the intermediate state of BTG transformation to PTC, remains unknown. Differences in tissue and serum protein profiles were reported in PTC patients with and without BTG background (8) indicative of differences in the underlying mechanisms between the two PTC subtypes and/or differences in their disease progression. This study aimed to evaluate gene mutation profiles to further understand the molecular mechanism underlying BTG, PTC without BTG background and PTC with BTG background. The presence of distinct tumour-related genetic profiles may be able to differentiate the two PTC subtypes in this cohort of patients.

## 2 Materials and methods

### 2.1 Subjects

This study was approved by the University of Malaya Medical Centre (UMMC)’s Medical Research Ethics Committee (MREC ID NO: 2019619-7540) in accordance with the ICH GCP guidelines and the Declaration of Helsinki. Written informed consent was obtained from all patients before the study was carried out.

The malignancy status of the patients was assessed through FNA cytology (FNAC) and was further confirmed by histopathological examination (HPE) of tissue specimens following partial or total thyroidectomy. All thyroid tissue specimens were placed in Allprotect tissue reagent (Qiagen, Hilden, Germany) at the time of retrieval and then stored at –80°C until further analysis. The patients were categorised into three groups based on the HPE reports: i) BTG (n = 9), ii) PTC without a BTG cytomorphological background (PTCa) (n = 8) and iii) PTC with a background of BTG (PTCb) (n = 5).

### 2.2 Genomic DNA extraction from thyroid tissue

Freshly excised thyroid tissue sample from each individual in the respective groups was submerged overnight in Allprotect tissue reagent (Qiagen, Hilden, Germany) at 4°C before storage at –80°C. Genomic DNA (gDNA) was extracted from the tissue samples using Qiagen AllPrep DNA/RNA/Protein Mini Kit (Qiagen, Hilden, Germany) according to the manufacturer’s protocol. The concentration and purity of the extracted gDNA were determined by Invitrogen Qubit dsDNA BR Assay kit (Thermo Fisher Scientific, Massachusetts, USA) on Qubit 2.0 Fluorometer, and by Thermo Scientific NanoDrop™ 2000c Spectrophotometer (Thermo Fisher Scientific, Massachusetts, USA), respectively. The gDNA integrity test was then performed using 1% agarose gel electrophoresis.

### 2.3 Whole-exome sequencing analysis

Genomic DNA (gDNA) samples of the patients; BTG (n = 9), PTCa (n = 8), and PTCb (n = 5) were sent to BGI Biotechnology Company (Shenzhen, China) for whole-exome sequencing (WES) analysis. The qualified gDNA was randomly fragmented into fragments with a range of 150-200 bp using the Adaptive Focused Acoustics^®^ (AFA^®^) technology of Covaris Ltd. The adapter-ligated templates were purified using the AgencourtAMPure Solid Phase Reversible Immobilisation (SPRI) beads and fragments with an insert size of about 200 bp were excised. Extracted adapter-ligated templates were then amplified by ligation-mediated polymerase chain reaction (LM-PCR), purified, and hybridised to the SureSelect Biotinylated RNA baits for enrichment and measured using the Agilent 2100 Bioanalyzer. The captured products were then circularised before rolling circle amplification (RCA) was used to produce DNA Nanoballs (DNBs). The captured libraries were then sequenced using the BGISEQ-500 sequencing platform and processed by BGISEQ base-calling Software with default parameters. The filtered WES data in FASTQ format were aligned to Reference Genome of human genome built 37 (http://hgdownload.cse.ucsc.edu/goldenPath/hg19/bigZips/) using Burrows-Wheeler Aligner (BWA) software. Genomic variations were detected by HaplotypeCaller of Genome Analysis Toolkit (GATK) (v3.6). The hard-filtering method was applied to obtain high confident variant calls. All the SNVs were then annotated using the SnpEff program (http://snpeff.sourceforge.net/). The WES results were validated by *BRAF* ^V600E^ mutation screening using PCR-direct DNA sequencing (9). Primers targeting the *BRAF* mutation were designed using Primer3 (https://primer3.ut.ee/). The primer sequence is as follows; Forward: 5’-CTCTTCATAATGCTTGCTCTGATAG-3’; Reverse: 5’-CCTCAATTCTTACCATCCAC-3’.

### 2.4 Variant filtering and principal component analysis

The variants with the following criteria were retained for further analyses: (i) minor allele frequency (MAF) ≤ 0.01 in the 1000 Genomes Project control database; and (ii) nonsynonymous and splice-site variants. Principal component analysis (PCA) was carried out on the retained SNVs using Python 3.6 software from a single computer workstation equipped with 3.1 GHz Dual-Core Intel Core i5 and 8 GB RAM. All the software and Python libraries used in the study work were from open sources. The following four variables of categorical data were used to represent an SNV molecular component chosen for the PCA: sample ID (e.g. patients 1, 2 etc), mutant genes (e.g. *AATF*), specific mutation (e.g. NM_012138:c.G872A:p.G291D) and genotype status (heterozygous/homozygous) as well as three classes of the disease type (BTG, PTCa and PTCb). One hot encoding was then run to convert the categorical variables into the numerical interpretation. A new dimension data of 22,427 rows and 22,850 columns were produced. All data were standardised in such a manner that it has mean as 0 and standard deviation as 1. During model training, the PCA algorithm was carried out to reduce high dimensional data into a minimum number of dimensions with 90% of the retained variance.

### 2.5 Database search of single nucleotide variants in tumour-related genes

The variants were filtered against two cancer-related gene panels from Oncology Knowledge Base (OncoKB) and Catalogue Of Somatic Mutation In Cancer (COSMIC) Cancer Gene Census databases (10, 11). The filtered variants were then screened through the Single Nucleotide Polymorphisms Database (dbSNP, http://www.ncbi.nlm.nih.gov/snp) and COSMIC database for identification of those that were novel. To determine PTC-specific SNVs, those that were identified in more than one PTC patients were further subjected to the Allele Frequency Aggregator (ALFA) project and the genome Aggregation Database (gnomAD) through Ensembl database (https://asia.ensembl.org/index.html) of which SNVs with MAF < 0.01 were retained. The PTC-specific SNVs were then analysed for their potential functional impact using Polymorphism Phenotype 2 (PolyPhen-2, http://genetics.bwh.harvard.edu/pph2/) and Sorting Intolerant from Tolerant (SIFT, https://sift.bii.a-star.edu.sg/). The variants predicted to be deleterious were further validated using Sanger sequencing method.

### 2.6 Protein-protein interaction network enrichment analysis

PPI enrichment analysis was carried out through Metascape tool (http://metascape.org) with the following databases: STRING, BioGrid, OmniPath, InWeb_IM9 using default parameters. Tumour-related genes that were found to be mutated in each disease group were used as the input gene sets. STRING (physical score > 0.132) and BioGrid were used to identify the protein-protein physical interactions. The Molecular Complex Detection (MCODE) algorithm was then applied to identify densely connected network components in the PPI analysis results. Pathway and process enrichment analyses were then carried out on the MCODE modules based on the GO Biological Processes and KEGG Pathway biological pathway databases.

## 3 Results

### 3.1 Nonsynonymous and splice-site variants identification and PCA analysis

**Figure 1:**
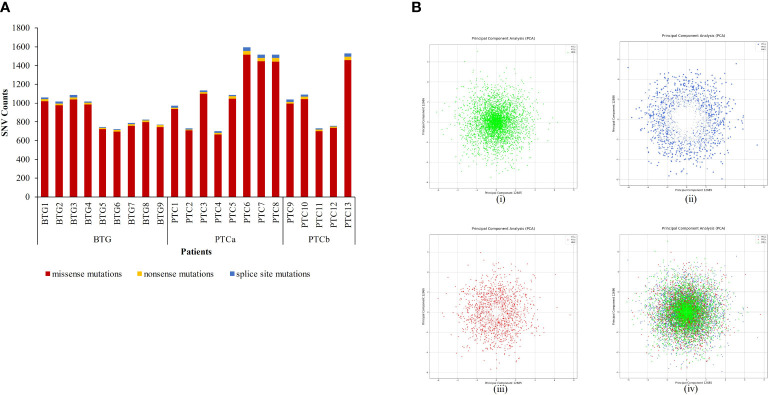
Analysis of nonsynonymous and splice-site single nucleotide variants (SNVs) identified through whole exome sequencing in patients with benign thyroid goitre (BTG), papillary thyroid cancer without BTG background (PTCa) and with BTG background (PTCb). **(A)** Nonsynonymous SNVs identified in each patient were filtered against 1000 Genome Projects (MAF ≤ 0.01). **(B)** Principal Component Analysis (PCA) of SNV profiles (gene, mutation, and genotype status) of the filtered nonsynonymous SNVs in (i) BTG, (ii) PTCa in relation to BTG, (iii) PTCb in relation to BTG and (iv) all disease groups.

A total of 15703 nonsynonymous and splice-site variants were identified in the three cohorts of patients (**Figure 1A**). PTCa patients had the highest average variant count (≈ 1156) followed by PTCb (≈ 1029) and BTG (893) patients. Missense mutation was found to be the most common type of variant type among all subjects. PCA plots of the filtered WES data for the three groups are shown in **Figure 1B**. In general, distinctive patterns of the four variables were observed for the BTG (**Figure 1B(i)**), PTCa (**Figure 1B(ii)**) and PTCb (**Figure 1B(iii)**) when the data were analysed separately. A similar distinctive pattern was observed when the datasets from the three disease groups were co-analysed. The variable profiles for PTCa showed a higher degree of dispersal compared to PTCb in relation to BTG. In addition, BTG, PTCa and PTCb showed some extent of similarities in their patterns represented by the shared area at the centre of the PCA plot [**Figure 1B(iv)**].

### 3.2 Analysis of mutated tumour-related genes in BTG, PTCa and PTCb

**Figure 2:**
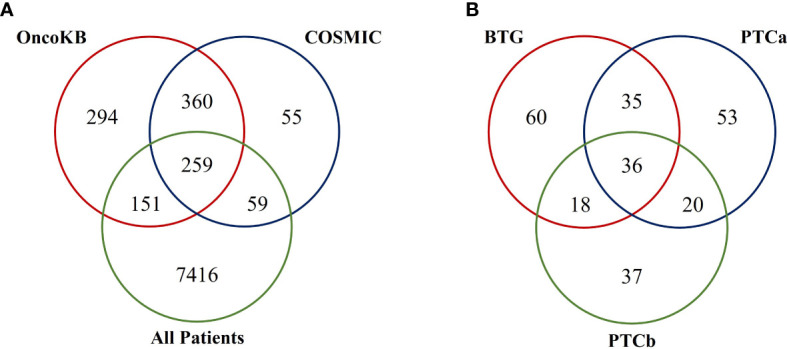
Venn diagrams illustrating findings from the database search of mutated tumour-related genes in this cohort of patients. **(A)** A total of 259 mutated tumour-related genes were listed in both the OncoKB and COSMIC databases. **(B)** Among the 259 mutated tumour-related genes, there were 36 genes found to be mutated in all three disease groups.

The 15703 nonsynonymous and splice-site variants were found in a total of 7875 genes in the three groups of patients. When the 7875 genes were filtered against OncoKB and COSMIC cancer gene census databases, a total of 259 tumour-related genes were identified (**Figure 2A**). OncoKB was able to identify more tumour-related genes in our cohort of patients (410 genes) compared to the COSMIC database (318 genes). Among the 259 mutated tumour-related genes, 149 were found in the BTG group, followed by 144 and 111 in the PTCa and PTCb groups, respectively (**Figure 2B**). Thirty-six tumour-related genes including *ALK*, *AR*, *BRCA2*, *FANCD2* and *CBL* were found to be mutated in all the three disease groups. An additional 35 genes including *ATR* and *IRS4* were found only in the BTG and PTCa. Another 18 tumour-related genes such as *NCOR2* and *PALB2* were found to be mutated in BTG and PTCb only. In addition, genes including *BRAF*, *DCTN1* and *RAD51B* were among the 20 tumour-related genes that were found to be mutated only in the PTCa and PTCb patients. The list of mutated tumour-related genes in each disease group is presented in **Supplementary Table 1. A** representative of the Sanger sequencing result of the *BRAF* ^V600E^ is shown in **Supplementary Figure 1**.

### 3.3 Protein-protein interaction network enrichment analysis of the mutated tumour-related genes found in BTG, PTCa and PTCb

**Figure 3:**
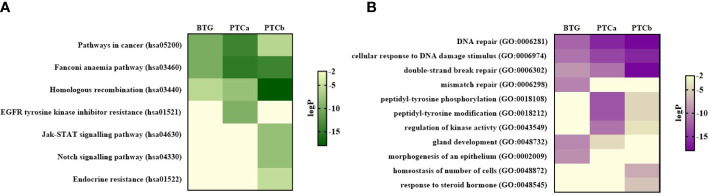
Protein-protein interaction (PPI) network analysis showing the top enriched pathways for the three disease groups based on the **(A)** KEGG database and **(B)** GO ontology database. Pathways with p-value < 0.01 (LogP < –2) was considered as significant. BTG, Benign thyroid goitre; PTCa, Papillary thyroid cancer without BTG background; PTCb, Papillary thyroid cancer with BTG background.

The top enriched KEGG ontology terms in the PPI networks of the three groups are shown in **Figure 3A**. “Pathways in cancer” (hsa05200), “Fanconi anaemia pathway” (hsa03460) and “Homologous recombination” (hsa03440) were significantly enriched in the three groups with “Pathways in cancer” and “Fanconi anaemia pathway” were the most enriched in PTCa while “Homologous recombination” was the most enriched in PTCb. “EGFR tyrosine kinase inhibitor resistance” (hsa01521) was exclusively enriched in PTCa while “Jak-STAT signalling pathway” (hsa04630), “Notch signalling pathway” (hsa04330) and “Endocrine resistance” (hsa01522) were only enriched in PTCb.

“DNA repair” (GO:0006281), “cellular response to DNA damage stimulus” (GO:0006974) and “double-strand break repair” (GO:0006302) were the enriched Gene Ontology (GO) terms shared among the three groups with the highest significance observed in PTCb followed by PTCa and BTG (**Figure 3B**). Three GO networks were only significantly enriched in the malignant groups namely “peptidyl-tyrosine phosphorylation” (GO:0018108), “peptidyl-tyrosine modification” (GO:0018212) and “regulation of kinase activity” (GO:0043549), with higher significance shown in PTCa compared to PTCb. “Mismatch repair” (GO:0006298), “gland development” (GO:0048732) and “morphogenesis of an epithelium” (GO:0002009) were highly enriched in the BTG group while “homeostasis of number of cells” (GO:0048872) and “response to steroid hormone” (GO:0048545) were only enriched in PTCb.

**Figure 4:**
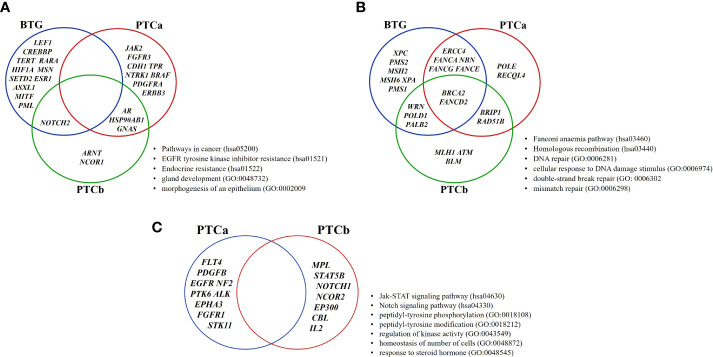
Distribution of genes of the enriched pathways in BTG, PTCa and PTCb. The enriched ontology terms are categorised according to the MCODE algorithm modules: **(A)** Cancer-related pathways and **(B)** DNA damage and repair-related pathways and **(C)** Signalling pathways. BTG, Benign thyroid goitre; PTCa, Papillary thyroid cancer without BTG background; PTCb, Papillary thyroid cancer with BTG background.

The enriched terms were categorised into three main modules: (a) Cancer-related pathways, (b) DNA damage and repair-related pathways, and (c) Signalling Pathways and the tumour-related genes that were involved in each module are shown in **Figure 4**. Although there was no shared gene among the three groups in the Cancer-related pathways, three genes were shared between the two malignant groups namely *AR*, *HSP90AB1* and *GNAS* (**Figure 4A**). *NOTCH2* was the only gene shared between BTG and PTCb. In the DNA damage and repair-related pathways, *BRCA2 (FANCD1)* and *FANCD2* were the only two genes shared by the three groups (**Figure 4B**). In addition to the two genes, *ERCC4, NBN, FANCA*, *FANCE* and *FANCG* were shared between BTG and PTCa; *WRN*, *POLD1* and *PALB2* were shared between BTG and PTCb groups while *BRIP1* and *RAD51B* were identified in PTCa and PTCb only. As shown in **Figure 4C**, none of the genes in the Signalling pathways were shared between the two malignant groups. BTG had none of the enriched term for this module.

### 3.4 Identification of potential molecular markers for PTC

A total of 108 SNVs were identified in more than one subjects. The complete list of the SNVs is presented in **Supplementary Table 2**. Comparative analysis against the ALFA project and gnomAD dataset through Ensembl database (https://asia.ensembl.org/index.html) identified 10 PTC-specific SNVs (**Table 1**). Four of the SNVs, *ATR* c.7817G>A (p.Arg2606Gln), *IRS4* c.605A>G (p.Lys202Arg), *PCM1* c.3520A>G (p.Thr1174Ala) and *TRRAP* c.8735G>C (p.Ser2912Thr) were only detected in PTCa. Two SNVs, *DCTN1* c.2786C>G (p.Ala929Gly) and *TRRAP* c.8735G>C (p.Ser2912Thr) were presumed novel as they could not be found in the dbSNP and COSMIC databases. *In silico* functional analysis through Polyphen2 predicted that the two novel mutations were “possibly damaging” while SIFT predicted that *DCTN1* c.2786C>G (p.Ala929Gly) and *TRRAP* c.8735G>C (p.Ser2912Thr) was “tolerated” and “deleterious” respectively. Another six previously reported SNVs namely *ARID1B* c.1181C>G (p.Ala394Gly), *BRAF* c.1799T>A (p.Val600Glu), *PDE4DIP* c.4073T>C (p.Ile1358Thr), *ATR* c.7817G>A (p.Arg2606Gln), *IRS4* c.605A>G (p.Lys202Arg), and *PCM1* c.3520A>G (p.Thr1174Ala) were also predicted to be deleterious. Although predicted to be of benign variants, *TET2* c.2440C>T (p.Arg814Cys) and *USP6* c.287G>T (p.Arg96Leu) showed PTC-specificity.

**Table 1:** Ten PTC-specific SNVs and their *in silico* functional impact predictions.

Gene	dbSNP	Transcript	Codon Change	Amino acid Change	SNV counts		PolyPhen2		SIFT	
					BTG	PTC	Score	Prediction	Score	Prediction
*ARID1B*	rs1778971493	NM_020732	c.1181C>G	p.Ala394Gly	0	3	0.766	Possibly Damaging	Not scored	
*DCTN1*	Novel	NM_004082	c.2786C>G	p.Ala929Gly	0	3	0.849	Possibly Damaging	0.370	Tolerated
*BRAF*	rs113488022	NM_004333	c.1799T>A	p.Val600Glu	0	3	0.923	Possibly Damaging	0.010	Deleterious
*TET2*	rs192553789	NM_001127208	c.2440C>T	p.Arg814Cys	0	2	0.034	Benign	0.120	Tolerated
*USP6*	rs113754955	NM_001304284	c.287G>T	p.Arg96Leu	0	2	0.069	Benign	0.193	Tolerated
*PDE4DIP*	rs2066638374	NM_001198834	c.4073T>C	p.Ile1358Thr	0	2	0.993	Probably Damaging	0.010	Deleterious
*ATR*	rs199948706	NM_001184	c.7817G>A	p.Arg2606Gln	0	2	0.935	Possibly Damaging	0.210	Tolerated
*IRS4*	rs753584516	NM_003604	c.605A>G	p.Lys202Arg	0	2	0.890	Possibly Damaging	0.280	Tolerated
*PCM1*	rs143680240	NM_006197	c.3520A>G	p.Thr1174Ala	0	2	0.005	Benign	0.018	Deleterious
*TRRAP*	Novel	NM_001244580	c.8735G>C	p.Ser2912Thr	0	2	0.976	Probably Damaging	0.020	Deleterious

BTG, Benign thyroid goitre; PTC, Papillary thyroid cancer. SNVs with PolyPhen2 score ranged from 0.15 to 1.0 are predicted to be possibly damaging, those with score > 0.85 are more confidently predicted to be damaging. SNVs with SIFT score < 0.05 are considered deleterious.

**Figure 5:**
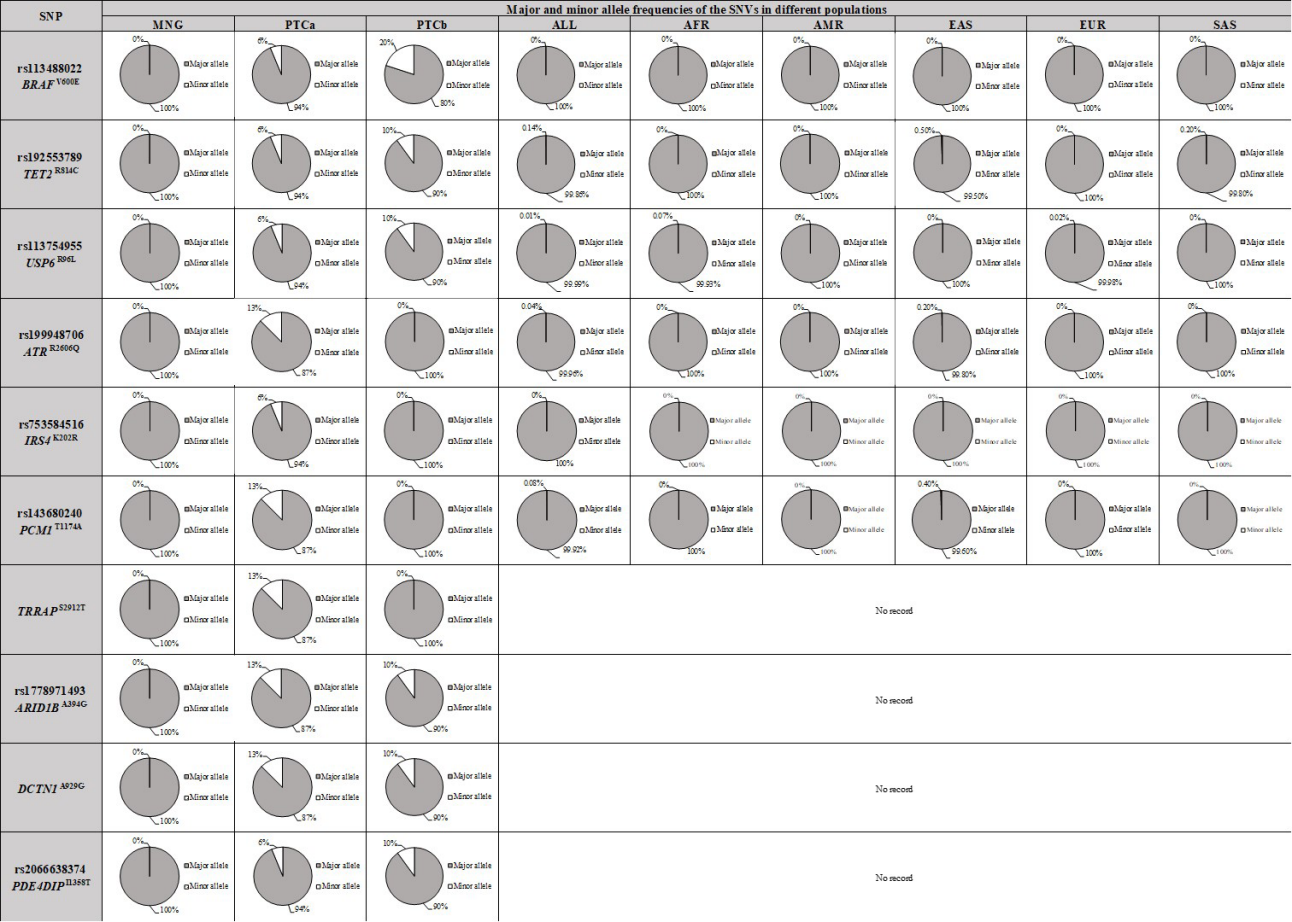
Comparison of allelic frequencies of ten PTC-specific SNPs in BTG, PTCa, PTCb and various populations. The data of different populations were obtained from Ensembl population genetic database (https://asia.ensembl.org/index.html). ALL, all populations; AFR, African; AMR, American; EAS, East Asian; EUR, European; SAS, South Asian. Major allele refers to the wildtype allele; minor allele refers to the mutant allele. BTG, Benign thyroid goitre; PTCa, Papillary thyroid cancer without BTG background; PTCb, Papillary thyroid cancer with BTG background.

Allelic frequency comparison of six SNPs: *BRAF* c.1799T>A (p.Val600Glu), *TET2* c.2440C>T (p.Arg814Cys), *USP6* c.287G>T (p.Arg96Leu), *ATR* c.7817G>A (p.Arg2606Gln), *IRS4* c.605A>G (p.Lys202Arg) and *PCM1* c.3520A>G (p.Thr1174Ala) with various normal populations is presented in **Figure 5**. The minor allele for the six PTC-specific SNVs were either totally absent (*BRAF* c.1799T>A (p.Val600Glu) and *IRS4* c.605A>G (p.Lys202Arg) or present in less than 1% in the world (ALL), African (AFR), American (AMR), East Asian (EAS), European (EUR) and South Asian (SAS) populations. Population genetic data for the other four SNVs; *ARID1B* c.1181C>G (p.Ala394Gly), *DCTN1* c.2786C>G (p.Ala929Gly), *PDE4DIP* c.4073T>C (p.Ile1358Thr) and *TRRAP* c.8735G>C (p.Ser2912Thr) were not available in the database for allelic frequency comparison to be made.

## 4 Discussion

To understand the underlying genetic alterations in PTCa and PTCb, WES data of the two groups were compared to those in BTG. WES is one of the NGS platforms that can detect variations in the protein-coding area of genes (exons), which account for approximately 3.09% of the whole genome (12). Its function and application to detect disease-causing mutations have been previously demonstrated in papillary thyroid cancer cases (13–16) and other clinical studies (17, 18).

In this study, nonsynonymous variants with MAF ≤ 1% were prioritised due to their direct impact on the functionality and the available tools to interrogate their pathogenic effects (19, 20). The three groups showed similarities in the type of variants of which missense variants being the most common, followed by splice-site and nonsense variants. However, among the three groups, PTCa had the highest total number of variants, followed by PTCb and BTG. DNA damage is common, and it is usually repaired by various DNA repair machineries. The equilibrium between the occurrence of DNA damage and repair, if skewed, leads to DNA damage accumulation, and might start the cancer onset (21). DNA damage accumulation might have contributed towards the higher number of variants in both the malignant PTCa and PTCb groups as compared to the BTG. The different average number of variants between PTCa and PTCb may indicate differences in the underlying disease progression mechanisms or differences in the phases/states during the transformation from the benign to malignant condition. Since the transformed WES data had superhigh dimensions, PCA was done to reduce the dimensionality to find distinct patterns within the data without losing its original information. Ninety percent of variance were set during the model training to get the best visualisation of the distinct patterns while retaining as much information in the datasets as possible (22). The 10% loss in information is to be expected during the dimensional reduction and other factors including duplication, less informative or the complex nature of the information (23). Distinctive profiles were also observed when the filtered WES data for PTCa and PTCb in relation to BTG, were further visualised through PCA plots. This may also point to differences in the underlying molecular mechanisms. Despite the distinctive profiles, some extent of similar patterns in PCA plots were also observed among the BTG, PTCa and PTCb. The pattern similarities could be interpreted that both PTCa and PTCb originate from BTG or PTCb could be an intermediate state in the BTG to PTCa transformation.

To further understand the similarities and differences in the underlying molecular mechanisms behind PTCa and PTCb in relation to BTG, the gene variants identified in all patients were subjected to comparative analyses against OncoKB and COSMIC cancer gene census databases followed by PPI pathway enrichment analyses. MCODE algorithm was applied to identify the densely connected network components in the PPI analysis. The three groups shared significant enrichment of the Cancer-related pathways and DNA damage repair-related pathways. Although no specific tumour-related gene was shared among the three groups, *NOTCH2* was shared between BTG and PTCb while *AR*, *HSP90AB1* and *GNAS* were shared between BTG and PTCa suggesting their possible roles in the BTG-to-PTCb and BTG-to-PTCa transformation, respectively. These observations also suggest that the mechanism of transformation from BTG to PTCa differs from that of BTG to PTCb. In general, the DNA damage and repair-related pathways were more significantly enriched in both PTCa and PTCb compared to BTG. “Fanconi anaemia pathway” was most enriched in PTCa while “Homologous recombination” and “mismatch repair” were most enriched in PTCb and BTG respectively. Notably, *BRCA2* and *FANCD2* were shared by three groups indicating that the two genes possibly playing key roles in triggering the transformation of BTG to both PTCa and PTCb. DNA lesions can be caused by either endogenous or exogenous agents, such as reactive oxygen species (ROS) produced during cell metabolism or ionising radiation (13). As such, cells are equipped with different DNA repair systems, including base excision repair (BER), nucleotide excision repair (NER), mismatch repair (MMR), homologous recombination (HR) and non-homologous end-joining (NHEJ) against different types of DNA damages and active throughout different cell cycle stages, maintaining our genome stability. Aberrant DNA repair might drive accumulation of the DNA damages, followed by cancer onset. DNA damage in thyroid disorders is common, due to the reliance on ROS in thyroid hormone biosynthesis (14, 15).

Fanconi anaemia (FA) pathway has been found to be important in DNA repair mechanisms (24, 25), although none of our patients had haematologic disorders. Based on the literatures, upon recognition and binding of FA core complex that comprises seven complementary groups (A, B, C, E, F, L and M) to the DNA lesions, the ubiquitination activated FANCD2-I will then localise to the damaged DNA loci. The FA pathway then regulates the subsequent repair processes including DNA replication, cell-cycle arrest, and DNA damage repair. BRCA2 is a recombination mediator that co-localises with ubiquitinated FANCD2 and facilitates the formation of Rad51 nucleofilaments, linking the FA pathways to homologous recombination (26). *BRCA2* mutation has been linked to many cancer types, including breast cancer, ovarian cancer, pancreatic cancer and glioblastoma (27–30). Generally, cancer patients with the *BRCA2* mutations were reported to have a more aggressive phenotype compared to those with *FANCD2* mutation (24). In this study, in addition to *BRCA2* and *FANCD2*, another three genes in the FA pathways, *FANCA*, *FANCE* and *FANCG*, were exclusively shared between BTG and PTCa indicating their importance in the transformation of BTG to PTCa with an aggressive phenotype.

In addition to *BRCA2* and *FANCD2, PALB2* is another gene node which was found in both BTG and PTCb. PALB2 has been reported to be indispensable to BRCA1 and BRCA2 in DNA repair mechanisms (31, 32). In contrast to FANCD2 monoubiquitination which has a mild impact on HR, BRCA2 activity is essential for DNA double-strand break (DSB) repair (24). *PALB2* and *BRCA*s genes have been well-reported due to their high prevalence in breast cancer where patients with *PALB2* gene mutation were found to have shorter survival years (33–35). It was also reported that approximately 49.1% of thyroid cancer patients were found to have breast tumours (36). *PALB2* codes for a protein that serves as a bridge protein between BRCA1 and BRCA2 proteins to form a complex that initiates HR of the DSB (37, 38). Mutations in PALB2 might lead to its BRCAs binding function loss, which leads to the impaired HR pathway, and the accumulation of DNA DSB lesions. The *ATM* gene found in PTCb’s PPI network further strengthens our speculation. *ATM* gene codes for the checkpoint kinase ataxia telangiectasia mutated, which activates the intra-S checkpoint in response to DSBs to arrest DNA replication, the possible loss of this kinase function might lead to further DNA lesions accumulation in the PTCb group. Further assessment on DSBs in PTCb with concomitant presence of breast cancer is thus warranted.

While there were degrees of similarities and differences in DNA damage repair-related pathways gene enrichment, in PTCa and PTCb, the “Signalling pathways” enriched network were distinctive in the two groups. Genomic instability has been suggested to be an early event in cancer development, where the consecutive genetic alterations that affect the normal cell cycle machinery might promote progression from a relatively benign proliferative cells lump to malignant tumour (39–41). Oncogenic mutations that lead to abnormal synthesis of receptors or ligands involved in signalling pathways, such as growth factor receptor tyrosine kinases (RTKs) and serine/threonine kinase, can cause hyperactive oncogenic signalling pathways and dysregulate cell cycle machinery (40). Signalling pathways related to tyrosine kinases were the most significantly enriched in PTCa while “Jak-STAT signalling pathway” and “Notch signalling pathway” were the only significantly enriched in PTCb. BTG was found to have none of the enriched term for this module. Oncogenic activation of the MAPK signalling cascade was found to be indispensable to the PTC development (31). The oncogenic activation of MAPK cascade upregulates the expression of the receptor tyrosine kinase and its binding to the ligands, such as growth factors, might lead to the oncogenic cell proliferation. However, the absence of this MCODE network in the PTCb group further revealed the different underlying mechanisms in the PTCa and PTCb disease progression. Jak-STAT and Notch signalling pathways were shown to be affected in the PTCb group. Jak-STAT signalling pathway has been reported to have tumour effects in PTC development (42). An additional study had also reported the potential role of affected STAT3 pathway and MAPK signalling cascade due to prolonged H_2_O_2_ insult in the thyroid gland and leading to the PTC progression (43, 44). The role of Notch signalling in cancer progression remains controversial, as it was found to be oncogenic and tumour suppressive in different studies. Increased Notch1 and Notch2 expression in PTC cases were found to be associated with more aggressive phenotypes (45). The upregulated Notch1 signalling pathway was linked to metastasis, increased tumour size, and led to a poor prognosis in PTC patients (45, 46). The affected Notch signalling pathway in the PTCb patients in the present study might result in the poor prognosis of PTCb where their presence of BTG cytomorphological structure is in the background. However, discordant findings were also reported (47, 48).

**Figure 6:**
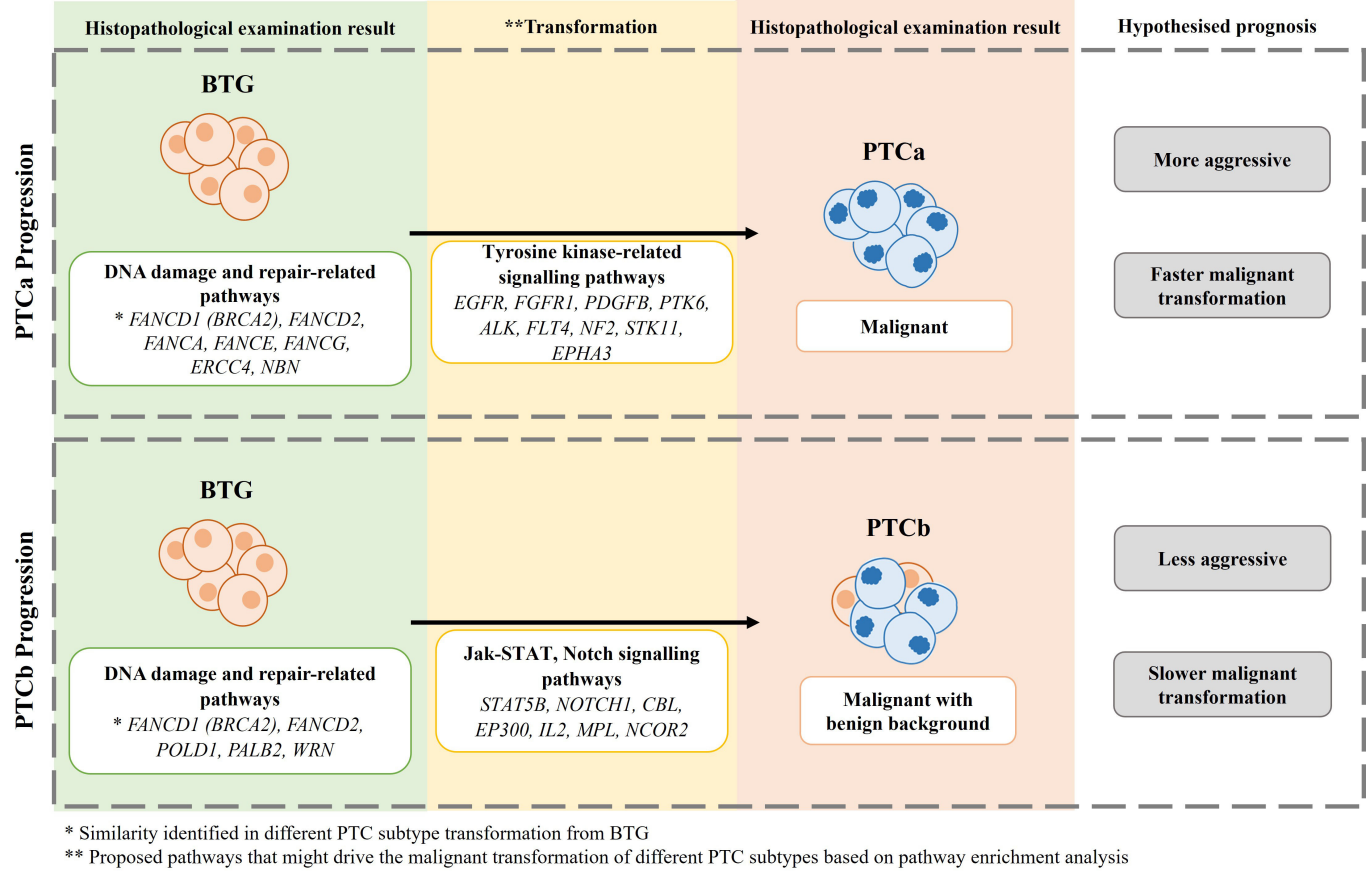
The proposed mechanism of BTG transformation to PTCa and PTCb in this cohort of patients.

Taken together, findings from the *in-silico* PPI network enrichment analysis suggest that erroneous DNA repair mechanism might be the primary cause of thyroid tumour development of BTG. Further oncogenic mutations that affect the signalling pathways then drive the transformation of PTCa and PTCb from BTG. The transformation of BTG to PTCa follows a different mechanism which involves a more aggressive tyrosine kinase-related pathways while the transformation of BTG to PTCb is linked to presumably a less aggressive JAK-STAT/Notch signalling pathways. Further confirmation on this is required. The difference in the signalling pathways will allow a more personalised therapeutic target for PTCa and PTCb. The proposed mechanism of transformation from BTG to PTCa and PTCb is summarised in **Figure 6**.

Through the WES sequencing analysis, there were ten SNVs showing PTC-specific characteristic where also predicted to be functionally deleterious in *in-silico* functional analysis *BRAF* c.1799T>A (p.Val600Glu) is one of ten SNVs that were found to be PTC-specific. *BRAF* ^V600E^ mutation, which was detected in approximately 60% of PTC cases, is associated with disease aggressiveness, recurrence and increased mortality (17, 18, 49–52). Its high prevalence in PTC patients has led to the suggestion of its potential use as a diagnostic and prognostic genetic biomarker (53–55). In the current study, *BRAF* ^V600E^ was confirmed to be PTC-specific. Its relatively low occurrence of 23.1% suggests that *BRAF* ^V600E^ is not a common underlying mutation in our cohort of patients. The prevalence of *BRAF* ^V600E^ mutation in PTC patients in Malaysia and in the rest of the Southeast Asia region remains largely unknown (56). While this mutation had been reported to be associated with intra-axial brain tumour patients in Malaysia (57), our study is the first to report the presence of this mutation in PTC patients in Malaysia. Further screening of the *BRAF* ^V600E^ mutation in a larger sample size will better reflect its prevalence in the Malaysian PTC population. While *BRAF* ^V600E^ was able to differentiate the BTG from the malignant PTC cases, it was unable to differentiate between PTCa and PTCb in this study.

Six SNPs, *BRAF* c.1799T>A (p.Val600Glu), *TET2* c.2440C>T (p.Arg814Cys), *USP6* c.287G>T (p.Arg96Leu), *ATR* c.7817G>A (p.Arg2606Gln), *IRS4* c.605A>G (p.Lys202Arg) and *PCM1* c.3520A>G (p.Thr1174Ala) were present in less than 1% of the world population as the African (AFR), American (AMR), East Asian (EAS), European (EUR) and South Asian (SAS) populations further suggesting that these were pathological instead of neutral mutations. The allelic frequency of another four SNVs {*ARID1B* c.1181C>G (p.Ala394Gly), *DCTN1* c.2786C>G (p.Ala929Gly), *PDE4DIP* c.4073T>C (p.Ile1358Thr) and *TRRAP* c.8735G>C (p.Ser2912Thr)} in the various populations were unknown due to data unavailability. While the ten SNVs were PTC-specific, four of the mutations; *ATR*, *IRS4*, *PCM1* and *TRRAP* were present only in PTCa. *ATR* encodes for serine/threonine-protein kinase ATR, is important for DNA damage sensors, activating DNA damage checkpoint against replication stress. The potential loss of function of ATR due to rs199948706 (p.Arg2606Gln) was suggested to increase the DNA damage, initiating the PTCa progression from BTG. Insulin receptor substrate 4 (IRS4) can induce hyperactivation of PI3K/AKT pathway and promote tumourigenesis, in the absence of insulin or other growth factors (56). *IRS4* mRNA is expressed in various human tissues including pituitary, thyroid, ovary, prostate, fibroblasts, however, with a low expression level (58). Upregulated *IRS4* was detected in various cancer cell lines, and high phosphatidylinositol triphosphate level was also identified (59, 60). *PCM1* codes for pericentriolar material 1, chromosomal aberrations of this gene have been linked to various malignancies. RET/PCM1 translocation had been identified in PTC patient, however its role in tumourigenesis is not fully elucidated. *TRRAP* mutation which was identified in *BRAF* wildtype PTC patients (61), plays an important role in the recruitment of histone acetyltransferase (HAT) complexes to the chromatin, regulating transcription and DNA repair. Its FATC domain can bind to Myc, regulating Myc oncogenic activities (62, 63). In this present study, the *TRRAP* p.S2912T mutation is located within the FAT domain, where the function and protein-protein interaction of this domain remains largely unknown. The ten SNVs may be useful as a diagnostic tool to determine malignancy status of thyroid nodules to complement the existing diagnostic methods pending further validation in a clinical setting and in a larger sample size.

In conclusion, distinctive gene mutation patterns were detected in BTG, PTCa and PTCb which corroborated the previous findings on protein profiles in similar cohort of patients. Based on the current study, it is hypothesised that *FANCD1* (*BRCA2*) and *FANCD2* along with other genes that encode for various components of the DNA damage and repair-related pathways, play a key role in the progression of BTG to PTC. Gene mutation patterns did not indicate that PTCb is the intermediate state of transformation of BTG to PTCa. Instead, PTCa and PTCb are subtypes that differ in the underlying molecular mechanisms involving tyrosine kinase-related signalling for the former and Jak-STAT and Notch signalling pathways for the latter. However, *in vitro* functional analysis would need to be carried out to further validate our speculations. The potential applications of the SNVs, especially the ten PTC-specific, in differentiating the benign from the PTC subtypes requires further validation in a larger sample size.

## Data availability statement

The data presented in the study are deposited in the Figshare repository, doi: 10.6084/m9.figshare.21714812.

## Ethics statement

The studies involving human participants were reviewed and approved by The University of Malaya Medical Centre (UMMC)’s Medical Research Ethics Committee (MREC ID NO: 2019619-7540) in accordance with the ICH GCP guidelines and the Declaration of Helsinki. The patients/participants provided their written informed consent to participate in this study.

## Author contributions

ZE participated in the study design, performed a major part of the experiments, analysed and interpreted the data, drafted the manuscript, prepared figures, and tables. MA performed part of the experiments, and reviewed manuscript drafts. KN performed surgeries, involved in specimen collection process, and contributed the clinical data of the patients and reviewed manuscript drafts. AA analysed the data, and reviewed manuscript drafts. NA collected samples, analysed the data, and reviewed drafts of the paper. NR analysed the data, and reviewed drafts of the paper. SJ conceived and designed the experiments, analysed, and interpreted the data, reviewed drafts, and critically revised the final manuscript. All authors contributed to the article and approved the submitted version.
