# Supplementary material for: Whole-exome sequencing and bioinformatic analyses revealed differences in gene mutation profiles in papillary thyroid cancer patients with and without benign thyroid goitre background
Source: Front Endocrinol (Lausanne). 2023 Jan 4;13:1039494. doi: 10.3389/fendo.2022.1039494 (PMC9846740; doi:10.3389/fendo.2022.1039494)
Supplement: Supplementary file 2 [file DataSheet_2.docx]

Supplementary Material

# Supplementary Data

Supplementary Material should be uploaded separately on submission. Please include any supplementary data, figures and/or tables. All supplementary files are deposited to FigShare for permanent storage and receive a DOI.

Supplementary material is not typeset so please ensure that all information is clearly presented, the appropriate caption is included in the file and not in the manuscript, and that the style conforms to the rest of the article. To avoid discrepancies between the published article and the supplementary material, please do not add the title, author list, affiliations or correspondence in the supplementary files.

# Supplementary Figures and Tables

For more information on Supplementary Material and for details on the different file types accepted, please see [here](http://home.frontiersin.org/about/author-guidelines#SupplementaryMaterial). Figures, tables, and images will be published under a Creative Commons CC-BY licence and permission must be obtained for use of copyrighted material from other sources (including re-published/adapted/modified/partial figures and images from the internet). It is the responsibility of the authors to acquire the licenses, to follow any citation instructions requested by third-party rights holders, and cover any supplementary charges.

## Supplementary Figures


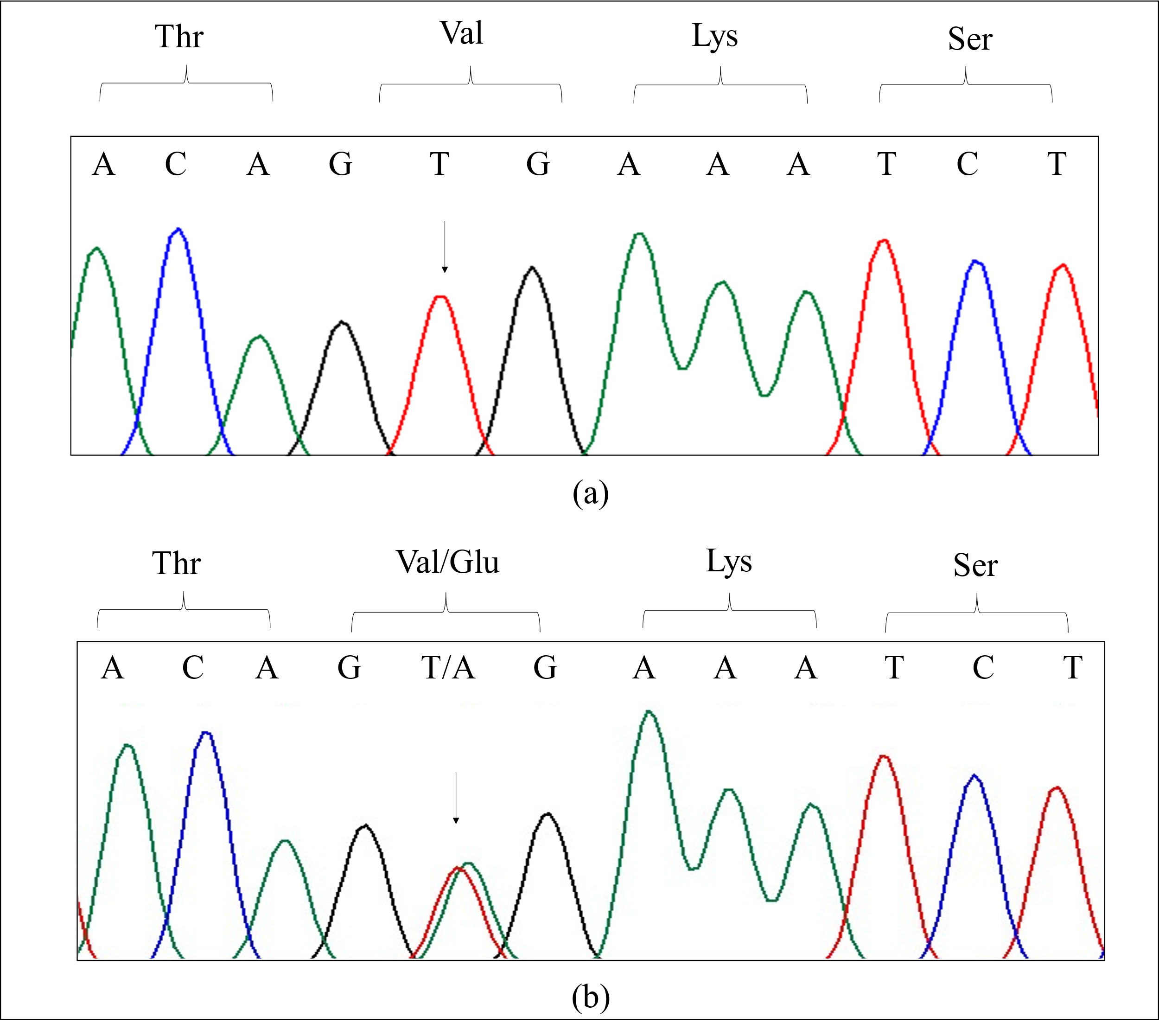


**Supplementary Figure 1.** **Detection of *BRAF*^V600E^ mutation (*BRAF* c.1799T>A) through Sanger sequencing** (a) Wildtype and (b) mutant *BRAF*^V600E^. The position of the c.1799T>A mutation is indicated by the arrow.
